# Lower Daily Walking Volume and Less Walking at Intermediate and Higher Cadences in Older Women with Clinician-Diagnosed Major Depressive Disorder: An Exploratory Cross-Sectional Study

**DOI:** 10.3390/healthcare14162530

**Published:** 2026-08-13

**Authors:** Jungeun Lee, Mansoo Ko

**Affiliations:** 1 Psychological, Health, and Learning Sciences Department, College of Education, University of Houston, Houston, TX 77204-5023, USA; jlee224@central.uh.edu; 2 Department of Physical Therapy and Rehabilitation Sciences, School of Health Professions, The University of Texas Medical Branch, 301 University Boulevard, Galveston, TX 77555-1144, USA

**Keywords:** major depressive disorder, older women, ambulatory activity, walking volume, cadence, wearable sensor, ROC analysis

## Abstract

**Background/Objectives:** Late-life depression is associated with reduced physical activity, but evidence is limited regarding objectively measured weekday walking volume and cadence distribution in older women with clinician-diagnosed major depressive disorder (MDD). This exploratory study compared daily walking between an MDD group and an age-comparable comparison group and examined the post hoc within-sample discrimination of two cadence-based ratios. **Methods:** Forty women aged 60–93 years (mean age, 71.8 ± 9.7 years) were classified into an MDD group (*n* = 20) and an age-comparable comparison group without a documented depressive-disorder diagnosis (*n* = 20). Current depressive symptoms were quantified using the Beck Depression Inventory-II (BDI-II), and free-living ambulatory activity was measured over three weekdays using StepWatch 3 monitors. Total instrumented-leg steps were recalculated as the sum of the low-, intermediate-, and higher-cadence step counts from the same three weekday summaries. The feasibility-based sample was analyzed using an exploratory MANOVA with SPSS-based assumption checks, Holm-adjusted follow-up tests, partial eta-squared effect sizes, and post hoc exploratory ROC analyses. **Results:** The multivariate group difference was significant (Pillai’s trace = 0.407, F (8,31) = 2.656, *p* = 0.024, partial η^2^ = 0.407). After Holm adjustment, the MDD group demonstrated lower total instrumented-leg steps, fewer intermediate- and higher-cadence walking minutes, fewer intermediate-cadence instrumented-leg steps, and a lower Peak Activity Index; the higher-cadence step-count difference did not remain significant after multiplicity adjustment. The Intermediate + Higher-Cadence Step Ratio showed an AUC of 0.828 (95% CI: 0.701–0.954), and the Intermediate + Higher-Cadence Minutes Ratio showed an AUC of 0.818 (95% CI: 0.684–0.951). **Conclusions:** In this exploratory cross-sectional sample, lower weekday walking volume and less walking at intermediate and higher cadences were observed in the MDD group. These sample-derived discriminatory findings are not diagnostic and require independent validation.

## 1. Introduction

Older adults often live with chronic conditions that may affect mobility, independence, and participation. Chronic disease can contribute to disability and functional decline [1], and depression may further complicate physical function, self-care, and engagement in usual activities.

Women comprise a large proportion of the older population because of their longer life expectancy [2]. Associations between depression, physical activity, and sedentary behavior have been reported in women and other adult samples [3,4]. Physical activity supports long-term health and survival in older adults [5], whereas late-life depression may coexist with poorer quality of life, reduced social participation, and lower engagement in health-promoting behaviors [6,7]. These considerations make objectively measured daily walking relevant for understanding routine activity engagement in older women.

Depression is commonly accompanied by reduced social and physical activity [6,7]. Daily walking is an accessible form of physical activity embedded in self-care, household tasks, errands, recreation, and community participation. In depression, walking may differ in amount, cadence, or sustained duration. Longitudinal evidence links physical activity with survival and depression with subsequent reductions in activity [5,8]. Other health-related factors, including diet and weight-related factors, may also interact with depression [9], and physical-activity intensity and type have been associated with depressive symptoms in older adults [10]. These observations place walking behavior within a multifactorial health context and support evaluating both walking volume and cadence distribution [10].

Older adults may face physical, environmental, and safety-related barriers to regular activity [11], and lower physical activity has been associated with depressive status among older women [12]. However, walking behavior is also shaped by mobility, health status, social context, transportation, and environmental opportunities. Recent systematic evidence identifies interacting physical, psychological, behavioral, social, environmental, and setting-specific determinants of physical activity and sedentary behavior in older adults [13]. Accordingly, observed group differences should not be interpreted as evidence of a simple causal pathway between depression and walking behavior.

Physical activity is related to long-term health, and depression may be followed by reductions in activity over time [5,8]. Although physical activity and sedentary behavior are associated with depression and physical-activity programs may improve depressive symptoms in some older residential populations [4,14], daily walking can occur both intentionally as exercise and incidentally during routine and community activities. Objective monitoring of walking volume and cadence can therefore characterize daily ambulatory patterns without relying solely on self-report.

Depression includes affective, behavioral, and somatic symptoms such as diminished interest, fatigue, and psychomotor change [15], which may reduce the capacity to initiate or sustain activity. Such differences may appear not only as lower walking volume but also as less walking at faster instrumented-leg cadences. Total steps reflect ambulatory volume, whereas cadence-band measures describe how recorded steps and active minutes are distributed across slower and faster walking. This rationale is consistent with behavioral activation models emphasizing reduced engagement in rewarding or goal-directed activities [16]. Walking volume and cadence distribution may therefore represent activity-derived characteristics associated with depression status, while remaining nonspecific and sensitive to physical and environmental influences.

Although objectively measured physical activity has been examined in relation to depression, two gaps remain. Self-reported physical activity is still common [17], and objective studies often involve nonclinical samples rather than participants with documented depressive disorders [18,19]. In addition, daily walking volume and cadence distribution have rarely been evaluated together in older women, and the within-sample discriminatory performance of cadence-based ratios remains uncertain. The present study addressed these gaps by combining clinician-documented MDD status with objective weekday measures of ambulatory volume, active minutes, cadence bands, and peak cadence. The post hoc exploratory ROC analyses were intended to describe within-sample group discrimination, not establish diagnostic validity.

Accordingly, the primary aim of this exploratory cross-sectional study was to compare objectively measured daily walking volume and cadence distribution between older women with clinician-diagnosed MDD and an age-comparable comparison group. A secondary exploratory aim was to evaluate the within-sample discriminatory performance of two cadence-based ratios using receiver operating characteristic (ROC) curve analysis. We hypothesized that the MDD group would demonstrate lower daily walking volume and less walking in the intermediate- and higher-cadence bands than the comparison group. We further hypothesized that the two cadence-based ratios would show exploratory discriminatory ability between the two preselected groups.

## 2. Methods

### 2.1. Study Design

This exploratory, observational, cross-sectional study was conducted in San Angelo and the surrounding Concho Valley, Texas, from January 2014 through at least August 2015; the complete participant-level monitoring-date log was not retained. The present report is based on an archived dataset from the completed study, with no additional participant contact or data collection. The study was approved by the Angelo State University Institutional Review Board (IRB #Lee-1213; 5 December 2013), was conducted in accordance with the Declaration of Helsinki, and all participants provided written informed consent before study procedures. Reporting was reviewed against the Strengthening the Reporting of Observational Studies in Epidemiology (STROBE) checklist for cross-sectional studies [20].

After eligibility confirmation and consent, participants completed demographic and clinical data collection and the Beck Depression Inventory-II (BDI-II) at enrollment. A StepWatch 3 monitor was then programmed and worn during three scheduled free-living weekdays; weekend days were intentionally excluded to standardize the sampled day type. Each participant contributed three included daily summaries (mean, 3.0 days), and participant-level walking variables were calculated as the mean of those three weekdays. These estimates therefore characterize the sampled weekdays rather than a complete seven-day pattern. The primary analysis compared eight walking outcomes between the preselected groups using exploratory MANOVA, followed by post hoc exploratory ROC analyses of two derived cadence-based ratios.

### 2.2. Participants

Women aged 60 years or older were recruited through separate pathways rather than from a single sampling frame. The MDD group was recruited through Mental Health and Mental Retardation for the Concho Valley (MHMR), clinician referral, and residential/senior-living settings; the comparison group was recruited through churches and other community settings, and residential/senior-living settings. Archived feasibility records indicated that the potentially eligible MHMR pool increased to 105 after the minimum age was lowered to 60 years. This was an eligibility pool rather than a formal screening log, and the numbers approached, assessed, excluded, declining participation, or otherwise not enrolled were not retained.

The MDD group required a documented clinical diagnosis of MDD made by a treating mental health clinician according to the criteria of the Diagnostic and Statistical Manual of Mental Disorders, Fourth Edition, Text Revision (DSM-IV-TR) [21]. Group classification was based on archived referral or enrollment documentation rather than a study-administered structured diagnostic interview. The archived records preserved reported duration since diagnosis but not the clinician’s specific professional credential, diagnostic instrument or interview, recency of the diagnostic assessment, current episode or remission status, antidepressant medication use, psychotherapy, or changes in treatment during monitoring. Comparison participants had no documented prior diagnosis of depressive disorder but did not undergo an equivalent structured diagnostic assessment. The archived materials did not retain a standardized medical-comorbidity screening instrument or systematic evaluation of neurological, orthopedic, cardiovascular, pulmonary, rheumatological, oncological, or other chronic conditions with uncontrolled symptoms; therefore, such conditions could not be reconstructed as additional exclusion criteria. The explicit exclusions documented in the available protocol were wheelchair confinement and cognitive disorder.

The BDI-II was administered at enrollment before monitor deployment to quantify current depressive symptoms; it was not used to establish eligibility, assign participants to groups, or confirm the clinical diagnosis. Depressive symptoms were not reassessed during the three monitoring weekdays; therefore, symptom stability across the monitoring period could not be verified. The protocol designated BDI-II evaluators as blinded to group status, although the success of blinding could not be verified from archived records; data analysts were not blinded because group labels were included in the analytic file. The comparison group was recruited to approximate the age distribution of the MDD group; no fixed one-to-one pairs, age strata, or matching caliper were used, and participants were analyzed as independent observations. Age, ethnicity, driving status, recruitment source, and duration since diagnosis were obtained from archived enrollment forms and recruitment records. All 40 enrolled participants (20 per group) contributed the data required for the present analyses and were included in the analytic sample.

### 2.3. Instruments

#### 2.3.1. Beck Depression Inventory-II (BDI-II)

The standard Beck Depression Inventory-II (BDI-II; second edition) [22] is a 21-item self-report measure of depressive symptom severity. Items are scored from 0 to 3, yielding a total score from 0 to 63; conventional ranges are 0–13 (minimal), 14–19 (mild), 20–28 (moderate), and 29–63 (severe). In this study, the BDI-II was used to quantify current depressive symptoms rather than to establish the clinical diagnosis, determine eligibility, or assign group status. The available archived materials did not document use of a translated or culturally adapted form. The protocol identified blinded study evaluators as overseeing administration at enrollment, but the professional credentials of individual evaluators were not retained. Item-level responses were not retained, so Cronbach’s α could not be calculated for the present sample.

The BDI-II has been evaluated in adolescent clinical samples [23], among adults living with HIV and family caregivers of children with chronic diseases [24,25], and in culturally or racially diverse populations [26,27]. It has also demonstrated strong psychometric properties in adults aged 50 years and older [28], supporting its use for symptom quantification in the present sample; however, these published properties do not substitute for a sample-specific internal-consistency estimate.

#### 2.3.2. StepWatch Activity Monitor (SAM)

The StepWatch 3 (SW3; Cyma Corporation, Mountlake Terrace, WA, USA) is an ankle-worn research-grade monitor for free-living ambulatory activity [29,30,31]. In the present study, the device recorded foot contacts of the instrumented leg, and data were summarized in one-minute epochs. The monitor did not provide real-time activity feedback. Previous studies reported high accuracy across varied gait patterns [30] and strong concurrent validity for stride counts in people with Parkinson disease or multiple sclerosis [32]. Because the device records foot contacts from one instrumented leg, its native output corresponds to instrumented-leg steps, equivalent to stride counts, rather than bilateral step counts [31,32]; counts were not doubled in the present analyses. The archived records did not preserve which ankle was instrumented or the detailed attachment procedure beyond ankle placement.

SW3 was released in 2004 and was the StepWatch generation available during the 2014–2015 data-collection period; subsequent work compared stride-count output across SW3, SW4, and SW5 [31]. The exact software and firmware version numbers were not preserved. Before monitoring, each device was programmed using the participant’s height and observed gait characteristics. During an observed walking trial, LED detections were compared with manually counted instrumented-leg foot contacts, and sensitivity was adjusted when necessary to avoid under- or overcounting. Participants were instructed to wear the monitor continuously, including during sleep, for three scheduled weekdays; weekends were intentionally excluded. All participants contributed three included daily summaries (mean, 3.0 days), and no StepWatch values used in the analyses were missing; therefore, no imputation was performed. A formal wear-time threshold, individual monitoring dates for seasonal analysis, bathing or aquatic-activity instructions, separate non-wear logs, and an algorithmic non-wear classification were not retained in the archived records.

Cadence was categorized from the archived StepWatch summary variables into mutually exclusive bands: low-cadence, 1–15 instrumented-leg steps/min; intermediate-cadence, 16–40 steps/min; and higher-cadence, ≥41 steps/min. These were operational device-summary categories and should not be interpreted as physiological exercise-intensity thresholds validated for older women. Wearable and smartphone-derived movement measures have been studied as research outcomes in other clinical populations [33], but that literature does not validate the specific cadence bands used here. The Peak Activity Index was defined as the average cadence of the 30 highest, nonconsecutive one-minute epochs within a day, expressed as instrumented-leg steps/min and averaged across included days [34]. Minutes with zero recorded steps could represent sitting, standing, sleep, device removal, or other non-ambulatory periods and were not interpreted as sedentary time [35].

To describe cadence distribution beyond total walking volume, two ratio variables were derived. Model 1, the Intermediate + Higher-Cadence Step Ratio, was calculated as the proportion of cadence-band instrumented-leg steps accumulated in the intermediate- and higher-cadence bands relative to the sum of steps accumulated across the low-, intermediate-, and higher-cadence bands:

$$Model\;1=\frac{Step\;Numbers\;Intermediate-Cadence+Step\;Numbers\;Higher-Cadence}{Cadence-Band\;Step\;Numbers}$$

Model 2, the Intermediate + Higher-Cadence Minutes Ratio, was calculated as the proportion of active walking minutes in the intermediate- and higher-cadence bands relative to the sum of minutes with at least one recorded instrumented-leg step:

$$Model\;2=\frac{Step\;Minutes\;Intermediate-Cadence+Step\;Minutes\;Higher-Cadence}{Active\;Cadence-Band\;Minutes}$$

These ratios described the proportion of recorded walking accumulated in the intermediate- and higher-cadence bands. An originally exported total-step field did not correspond to the three-weekday cadence-band summaries and may have summarized a different monitoring interval. To ensure that all walking-volume variables referred to the same three included weekdays, that field was not used in the present analysis. Total instrumented-leg steps were recalculated for each participant as the sum of the low-, intermediate-, and higher-cadence step counts. Consequently, the total-step outcome and the Model 1 denominator were derived from the same three cadence-band counts. Both ratios were entered into the exploratory ROC analyses.

### 2.4. Statistical Analysis

All analyses were conducted using IBM SPSS Statistics version 28 (IBM Corp., Armonk, NY, USA), with statistical significance set at α = 0.05 using two-tailed tests. Numerical participant characteristics are reported as mean ± standard deviation (minimum–maximum), and categorical characteristics as *n* (%). Age and BDI-II scores were compared descriptively using Welch’s independent-samples *t* tests; ethnicity and recruitment source were evaluated using two-sided Fisher–Freeman–Halton exact tests, and driving status was evaluated using a two-sided Fisher’s exact test. These participant-characteristic comparisons were exploratory and were not adjusted for multiplicity.

The sample size was determined by recruitment feasibility (20 participants per group); no a priori power analysis was performed, and all analyses were considered exploratory. MANOVA was selected to evaluate a joint group difference across eight related ambulatory outcomes rather than treating each outcome as an independent primary test. The eight dependent variables were (1) total instrumented-leg steps, (2) higher-cadence walking minutes, (3) intermediate-cadence walking minutes, (4) low-cadence walking minutes, (5) instrumented-leg steps accumulated in the higher-cadence band, (6) instrumented-leg steps accumulated in the intermediate-cadence band, (7) instrumented-leg steps accumulated in the low-cadence band, and (8) Peak Activity Index. Total steps were algebraically related to the three cadence-band step counts in the original units but were retained because overall walking volume and cadence distribution addressed distinct descriptive questions; the resulting redundancy was evaluated and reported. Log10(x + 1) transformations were applied to variables 1–7, whereas the Peak Activity Index was analyzed without transformation. Univariate distributions were examined using Shapiro–Wilk tests and Q–Q plots; covariance-matrix homogeneity using Box’s M test; equality of variances using Levene’s tests; redundancy using Pearson correlations; and multivariate outliers using group-specific Mahalanobis distances. Because several transformed distributions remained non-normal, Box’s M suggested covariance differences, and the outcomes were highly correlated, Pillai’s trace was used as the primary omnibus statistic. All observations were retained because no Mahalanobis distance exceeded the prespecified χ^2^(8, 0.001) cutoff. Follow-up *p* values were adjusted across the eight outcomes using the Holm step-down procedure implemented in IBM SPSS Statistics version (IBM Corp., Armonk, NY, USA) syntax, and partial eta-squared was reported from the SPSS GLM output. Descriptive values are presented in the original units, whereas inferential tests for variables 1–7 were conducted using transformed values.

The two cadence-based ratios, ROC analyses, and cutoff selection were not specified in the original protocol and were therefore labeled post hoc and exploratory. ROC curve analysis evaluated within-sample discrimination of the Intermediate + Higher-Cadence Step Ratio and Intermediate + Higher-Cadence Minutes Ratio between the two preselected groups; it was not considered diagnostic validation. The MDD group was specified as the positive state, and lower ratio values indicated a more positive result. For each ratio, the AUC, nonparametric 95% confidence interval, *p* value, and coordinate table were obtained using the SPSS ROC procedure. The cutoff maximizing the Kolmogorov–Smirnov statistic (sensitivity + specificity − 1) was selected; when multiple cutoffs produced the same maximum statistic, the cutoff with greater sensitivity was selected. Sensitivity, specificity, positive predictive value (PPV), negative predictive value (NPV), accuracy, and likelihood ratios were calculated from 2 × 2 tables. Wilson 95% confidence intervals for sensitivity, specificity, PPV, NPV, and accuracy and log-method intervals for likelihood ratios were calculated using SPSS syntax. No bootstrap, cross-validation, or other internal-validation procedure was performed; all cutoff and classification estimates are sample-derived and require external validation.

## 3. Results

### 3.1. Participant Characteristics

All 40 enrolled participants were analyzed. Age (Welch t [37.8] = 0.85, *p* = 0.403), ethnicity (Fisher–Freeman–Halton exact *p* = 0.197), and driving status (Fisher’s exact *p* = 0.127) did not differ significantly between groups. BDI-II scores were higher in the MDD group (Welch t [28.0] = 8.63, *p* < 0.001), and recruitment source differed (Fisher–Freeman–Halton exact *p* < 0.001), reflecting the separate recruitment pathways. Comparison-group BDI-II scores ranged from 0 to 14; one participant scored 14, the lower boundary of the conventional mild range. Group assignment was based on the absence of a documented depressive-disorder diagnosis rather than the complete absence of depressive symptoms. Table 1 summarizes the sample; these exploratory comparisons do not establish group equivalence.

Numerical variables are presented as mean ± standard deviation (minimum–maximum), and categorical variables as *n* (%). *p* values were obtained using Welch’s independent-samples *t* tests (age and BDI-II score), a two-sided Fisher’s exact test (driving status), or two-sided Fisher–Freeman–Halton exact tests (ethnicity and recruitment source). Comparisons were exploratory and were not adjusted for multiplicity. No *p* value was calculated for duration since MDD diagnosis because it was not applicable to the comparison group. No values shown were missing. BDI-II = Beck Depression Inventory-II; MDD = major depressive disorder. Recruitment source was recorded but should not be interpreted as participants’ living setting. The comparison group had no documented depressive-disorder diagnosis but was not defined as symptom-free.

### 3.2. Daily Walking Activity

**Figure 1:**
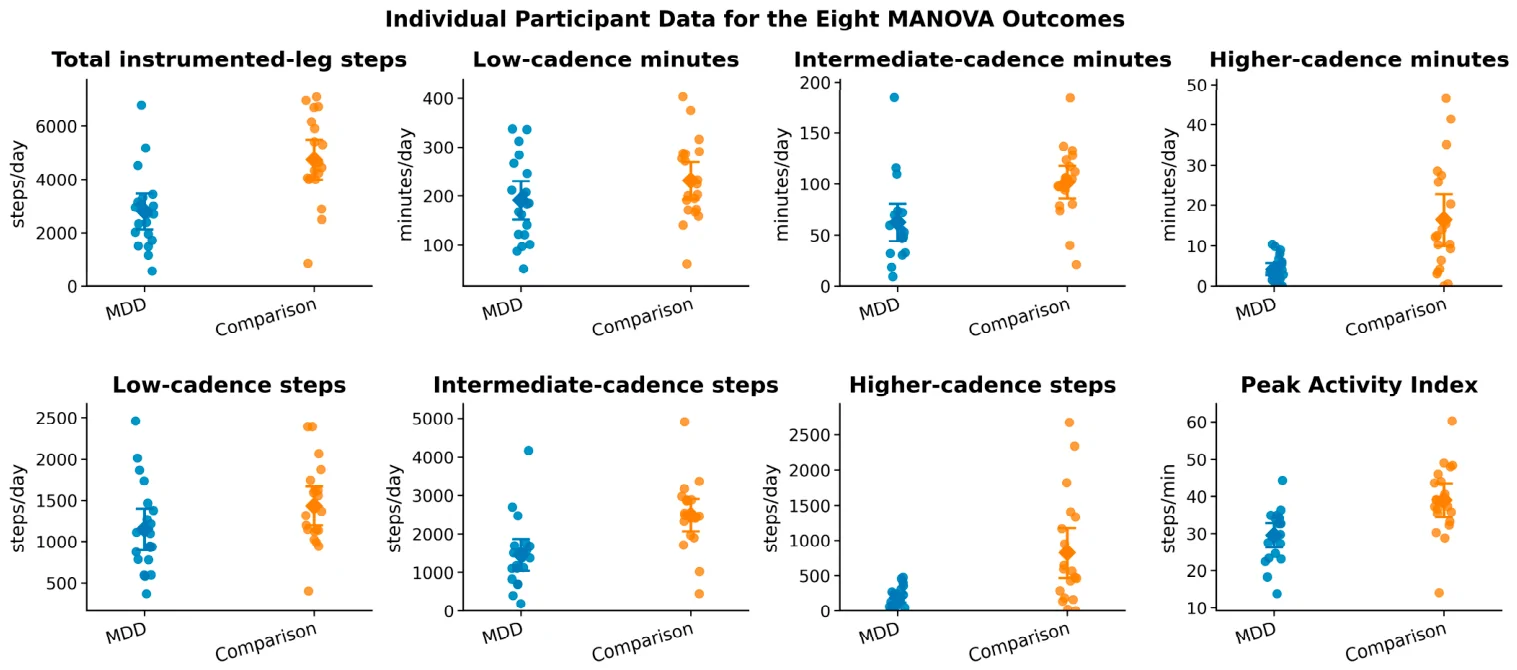
Individual participant values for the eight MANOVA outcomes. Blue symbols represent the MDD group, and orange symbols represent the comparison group. Diamonds and error bars represent group means and 95% confidence intervals. StepWatch counts represent instrumented-leg steps (equivalent to strides). Cadence-band labels are descriptive operational categories, not physiological exercise-intensity thresholds. Total instrumented-leg steps equal the sum of the three cadence-band step counts. Values are shown in the original units; inferential tests for the seven count/minute outcomes used log10(x + 1)-transformed values, and Holm-adjusted results and partial eta-squared effect sizes are reported in Table 2.

Assumption checks indicated non-normality in several transformed outcomes and some covariance heterogeneity. Six of the 16 group-specific distributions of the transformed outcomes remained non-normal by Shapiro–Wilk tests, consistent with the Q–Q plots. Box’s M was 72.24 (approximate F [36,4858.85] = 1.549, *p* = 0.019), whereas Levene’s tests were nonsignificant for all eight outcomes (*p* = 0.220–0.947). Pairwise correlations among transformed outcomes ranged from r = 0.519 to 0.997, and five correlations exceeded 0.90, indicating substantial redundancy. No participant exceeded the prespecified group-specific Mahalanobis-distance cutoff of χ^2^(8, 0.001) = 26.12 (maximum = 17.83). No values were missing for the eight analytic outcomes. Given these findings, Pillai’s trace was used as the primary multivariate test. The multivariate group difference was significant (Pillai’s trace = 0.407, F (8,31) = 2.656, *p* = 0.024, partial η^2^ = 0.407). After Holm adjustment, significant differences remained for total instrumented-leg steps (adjusted *p* = 0.007), higher-cadence walking minutes (adjusted *p* = 0.007), intermediate-cadence walking minutes (adjusted *p* = 0.008), intermediate-cadence instrumented-leg steps (adjusted *p* = 0.008), and Peak Activity Index (adjusted *p* = 0.007). The higher-cadence step-count comparison did not remain significant after correction (adjusted *p* = 0.053), and low-cadence minutes and steps were not significant (both adjusted *p* = 0.142). Descriptive statistics, unadjusted and Holm-adjusted *p* values, and partial eta-squared effect sizes are reported in Table 2. Individual participant data for the eight MANOVA outcomes are shown in Figure 1.

### 3.3. Exploratory Discriminatory Performance of Cadence-Based Ratios

Because the ratios and ROC analyses were post hoc, all estimates are presented as exploratory within-sample discrimination rather than diagnostic accuracy.

**Figure 2:**
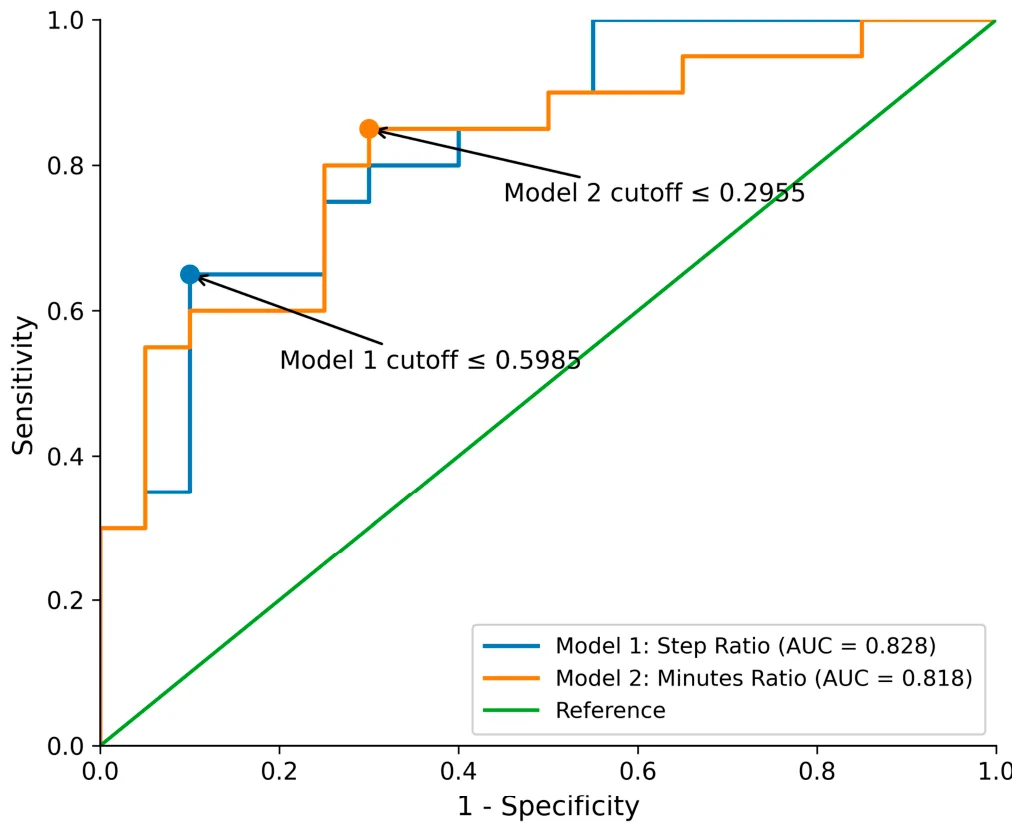
Receiver operating characteristic curves for the Intermediate + Higher-Cadence Step Ratio and Intermediate + Higher-Cadence Minutes Ratio. Markers indicate the sample-derived selected cutoffs (Model 1, ≤0.5985; Model 2, ≤0.2955). AUC and classification confidence intervals are reported in Table 3.

Model 1 (Intermediate + Higher-Cadence Step Ratio) demonstrated an AUC of 0.828 (95% CI: 0.701–0.954, *p* < 0.001). The maximum Kolmogorov–Smirnov statistic selected a cutoff of ≤0.5985, yielding 65.0% sensitivity, 90.0% specificity, and 77.5% apparent accuracy (31/40; 95% CI: 62.5–87.7). Model 2 (Intermediate + Higher-Cadence Minutes Ratio) demonstrated an AUC of 0.818 (95% CI: 0.684–0.951, *p* < 0.001). Two cutoffs produced the same maximum Kolmogorov–Smirnov statistic; the cutoff with greater sensitivity, ≤0.2955, was selected, yielding 85.0% sensitivity, 70.0% specificity, and 77.5% apparent accuracy (31/40; 95% CI: 62.5–87.7). The two ratios were strongly correlated (r = 0.904, *p* < 0.001). Complete same-sample classification estimates and confidence intervals are shown in Table 3. These cutoffs and performance estimates were derived and evaluated in the same sample; no internal-validation procedure was performed. The ROC curves for both cadence-based ratios are shown in Figure 2.

## 4. Discussion

### 4.1. Interpretation of Main Findings

The present study compared objectively measured weekday walking volume and cadence distribution between older women with clinician-diagnosed MDD and an age-comparable comparison group. The exploratory MANOVA indicated an overall group difference, although assumption diagnostics demonstrated non-normality, some covariance heterogeneity, and substantial redundancy among several outcomes. After Holm adjustment, the MDD group demonstrated lower total instrumented-leg steps, fewer intermediate- and higher-cadence walking minutes, fewer intermediate-cadence instrumented-leg steps, and a lower Peak Activity Index. The higher-cadence step-count comparison did not remain significant after multiplicity correction, and low-cadence variables did not differ significantly. The two cadence-based ratios showed post hoc within-sample discrimination, but their performance estimates were derived and evaluated in the same sample.

The lower weekday walking volume observed in the MDD group is consistent with prior work showing that depression and depressive symptoms are associated with reduced physical activity in older adults and in individuals with MDD [36,37]. Total instrumented-leg steps represent ambulatory activity completed during the sampled weekdays. The observed difference may reflect mobility, physical function, transportation, living environment, opportunities for walking, depression-related factors, or other unmeasured determinants. Recent systematic evidence supports a broader framework in which physical health and well-being, psychological and behavioral factors, social and cultural context, environmental accessibility, and implementation setting interact to shape physical activity in older adults [13]. The difference cannot be attributed to depression alone because these potential determinants were not comprehensively measured. In addition, StepWatch recorded ambulatory activity only; the results should not be interpreted as direct evidence of sedentary time or as a measure of all forms of physical activity.

Group differences were more pronounced in the intermediate- and higher-cadence bands than in the low-cadence band. This pattern is broadly consistent with objective-monitoring studies showing associations between moderate-to-vigorous physical activity and fewer depressive symptoms and reduced physical activity among adults with major depression [19,37,38]. The intermediate- and higher-cadence bands may reflect faster instrumented-leg cadence or more sustained walking than the low-cadence band. Cadence-based research supports steps per minute as a practical indicator of ambulatory intensity [34]; however, cadence does not directly measure metabolic demand, perceived exertion, or cardiorespiratory intensity. The StepWatch bands were not validated as physiological moderate- or vigorous-intensity thresholds for this sample. Accordingly, the present findings should be interpreted as differences in free-living cadence distribution, not direct differences in exercise intensity.

The cadence-band differences may also reflect variation in mobility demands, daily routines, environmental opportunities, or social participation. Prior studies have linked social isolation, interventions that jointly promote social participation and physical activity, and social support with physical activity in older adults [39,40,41]. However, the present StepWatch data cannot determine walking location, purpose, social contact, or living setting. These possibilities are hypotheses for future study rather than explanations established by the current data.

The ROC analyses were post hoc and showed exploratory within-sample discrimination between two preselected groups. The Step Ratio had slightly higher AUC and specificity, whereas the Minutes Ratio had higher sensitivity; both produced the same apparent accuracy. The ratios were strongly correlated and should not be interpreted as complementary sequential tests. Because no bootstrap, cross-validation, or independent validation was performed, the AUCs, cutoffs, and classification estimates may be optimistic. The fixed 50% prevalence created by the two-group design also limits interpretation of PPV and NPV. The ratios should therefore be viewed as exploratory activity-derived indicators, not screening, confirmation, or diagnostic tools. Independent validation in a representative prospective cohort is required.

Prior work has linked lifestyle activities with late-life depressive symptoms [42], social participation with broader aging-related outcomes [43], and sedentary behavior with adverse health outcomes in older adults [44]. None of these constructs was directly measured in the present study. In particular, periods without recorded steps could include sitting, standing, sleep, transportation, or non-wear and cannot be interpreted as sedentary time.

### 4.2. Research and Clinical Interpretation

Free-living walking volume and cadence distribution may eventually provide adjunctive contextual information, but the present cross-sectional findings do not establish screening, early detection, treatment monitoring, or device equivalence. Wearable and smartphone studies show that activity and mobility data can be captured outside the clinic [45,46,47]; however, transferability from SW3 to smartphones, wrist-worn devices, or newer StepWatch generations was not evaluated. Prospective within-person studies are needed to determine whether sustained changes in an individual’s usual walking add information beyond validated symptom measures and clinical assessment. Until then, the sample-derived cutoffs should not guide diagnosis or treatment, and physical-activity planning should remain individualized to mobility, medical status, preferences, and environment [13].

### 4.3. Limitations and Future Research

Several limitations are central to interpretation. First, the cross-sectional design cannot establish temporal direction or causality. The small feasibility sample (*n* = 40) included only women from one region and was recruited through separate pathways without a single sampling frame, creating potential selection and referral bias and limiting generalizability. Second, MANOVA assumptions were not fully satisfied: several transformed outcomes remained non-normal, Box’s M suggested covariance differences, and several correlations exceeded 0.90. Pillai’s trace and Holm-adjusted follow-up tests improve transparency but do not remove this uncertainty. Third, living arrangement, healthcare access, physical function, socioeconomic circumstances, comorbidities, pain, frailty, falls, mobility-aid use, medication and treatment exposure, cognition, smoking, alcohol use, and walking opportunities were not systematically retained; adjusted analyses were therefore not feasible [13]. Fourth, monitoring covered only three weekdays. Individual dates, season and weather, formal wear-time criteria, bathing or aquatic-activity instructions, and non-wear logs were unavailable, and the study did not measure weekend activity, non-ambulatory physical activity, posture, or sedentary behavior. Fifth, the cadence bands were operational device-summary categories with uncertain physiological meaning. The originally exported total-step field did not correspond to the three-weekday cadence-band summaries and may have represented a different monitoring interval; because raw minute-level files were unavailable, total steps were reconstructed from the three archived cadence-band step counts. Sixth, the post hoc ROC ratios and cutoffs were derived and evaluated in the same small sample without internal or external validation; the fixed 50% prevalence limits PPV and NPV, and transferability to consumer devices was not established. Finally, the 2014–2015 dataset predates current diagnostic practices, treatment patterns, and wearable technology. The findings should therefore be considered preliminary and hypothesis-generating.

The study provides preliminary evidence that weekday walking volume and cadence distribution differed between older women with clinician-diagnosed MDD and an age-comparable comparison group. Strengths include objective free-living monitoring, analysis of both volume and cadence distribution, transparent SPSS-based assumption checks, multiplicity-adjusted follow-up tests, partial eta-squared effect-size reporting, and explicit acknowledgment of data and validation limitations. Future studies should use a common source population, standardized diagnostic assessment, complete recruitment and wear-time logs, prespecified outcomes, comprehensive covariates, retained raw minute-level data, and an independent validation sample.

Prospective repeated-measures studies should test whether weekday and weekend walking volume and cadence distribution change with depressive symptoms, treatment response, physical function, or community participation and whether they add information beyond established clinical measures. Any clinical application would require representative recruitment, standardized diagnosis, cross-device validity, prespecified thresholds, assessment of classification harms, and independent validation.

### 4.4. Conclusions

In this exploratory cross-sectional sample, older women with clinician-diagnosed MDD demonstrated lower weekday walking volume, fewer intermediate- and higher-cadence walking minutes, fewer intermediate-cadence instrumented-leg steps, and lower peak free-living cadence than the comparison group after multiplicity adjustment. These findings describe sample-specific differences in ambulatory volume and cadence distribution; they do not establish causality, screening, or diagnostic utility. The post hoc ratios and sample-derived cutoffs require confirmation in larger prospective studies with comprehensive clinical covariates, prespecified analyses, raw-data quality controls, and independent validation.

## Figures and Tables

**Table 1:** Available Participant Characteristics.

Characteristic	MDD Group (*n* = 20)	Comparison Group (*n* = 20)	*p* Value
Numerical variables, mean ± standard deviation (minimum–maximum)			
Age, years	73.1 ± 9.4 (61–90)	70.5 ± 10.0 (60–93)	0.403
BDI-II score	22.0 ± 8.2 (14–39)	4.3 ± 4.1 (0–14)	<0.001
Duration since MDD diagnosis, years	14.7 ± 13.1 (2–50)	Not applicable	-
Categorical variables, *n* (%)			
Ethnicity	White: 18 (90.0%) Hispanic: 1 (5.0%) Black: 1 (5.0%)	White: 16 (80.0%) Asian: 3 (15.0%) Native American: 1 (5.0%)	0.197
Driving status	Drives: 13 (65.0%) Does not drive: 7 (35.0%)	Drives: 18 (90.0%) Does not drive: 2 (10.0%)	0.127
Recruitment source	Mental health agency: 8 (40.0%) Clinician referral: 1 (5.0%) Residential/senior-living: 11 (55.0%) Church/community: 0 (0.0%)	Mental health agency: 0 (0.0%) Clinician referral: 2 (10.0%) Residential/senior-living: 5 (25.0%) Church/community: 13 (65.0%)	<0.001

**Table 2:** Follow-Up Comparisons and Partial Eta-Squared Effect Sizes for the Eight MANOVA Outcomes.

Outcome	MDD Mean ± SD	Comparison Mean ± SD	F (1,38)	*p*	Holm-Adjusted *p*	Partial η^2^
Total instrumented-leg steps	2795.0 ± 1443.1	4749.1 ± 1603.2	12.554	0.001	0.007	0.248
Higher-cadence walking minutes	4.2 ± 3.3	16.4 ± 13.7	13.153	0.001	0.007	0.257
Intermediate-cadence walking minutes	62.2 ± 38.9	101.8 ± 34.7	11.050	0.002	0.008	0.225
Low-cadence walking minutes	191.2 ± 84.7	231.3 ± 82.7	2.423	0.128	0.142	0.060
Higher-cadence instrumented-leg steps	190.6 ± 148.4	824.5 ± 758.4	6.156	0.018	0.053	0.139
Intermediate-cadence instrumented-leg steps	1454.1 ± 887.2	2491.1 ± 900.2	11.539	0.002	0.008	0.233
Low-cadence instrumented-leg steps	1150.3 ± 539.7	1433.5 ± 498.6	3.452	0.071	0.142	0.083
Peak Activity Index	29.6 ± 7.1	39.0 ± 9.5	12.500	0.001	0.007	0.248

Note: Descriptive values are reported in the original units; F tests for variables 1–7 used log10(x + 1)-transformed values. Holm-adjusted *p* values were calculated from the eight SPSS-generated univariate *p* values using SPSS syntax. Partial η^2^ values were obtained from the SPSS GLM output. Total instrumented-leg steps were calculated as the sum of the low-, intermediate-, and higher-cadence step counts from the same three weekday summaries.

**Table 3:** Exploratory Within-Sample Performance of the Two Cadence-Based Ratios.

Measure	Model 1	Model 2
AUC (95% CI)	0.828 (0.701–0.954)	0.818 (0.684–0.951)
Cutoff	≤0.5985	≤0.2955
Sensitivity, % (95% CI)	13/20, 65.0 (43.3–81.9)	17/20, 85.0 (64.0–94.8)
Specificity, % (95% CI)	18/20, 90.0 (69.9–97.2)	14/20, 70.0 (48.1–85.5)
Accuracy, % (95% CI)	31/40, 77.5 (62.5–87.7)	31/40, 77.5 (62.5–87.7)
PPV, % (95% CI)	13/15, 86.7 (62.1–96.3)	17/23, 73.9 (53.5–87.5)
NPV, % (95% CI)	18/25, 72.0 (52.4–85.7)	14/17, 82.4 (59.0–93.8)
LR+ (95% CI)	6.50 (1.68–25.16)	2.83 (1.42–5.67)
LR− (95% CI)	0.39 (0.21–0.72)	0.21 (0.07–0.63)

AUC = area under the receiver operating characteristic curve; CI = confidence interval; PPV = positive predictive value; NPV = negative predictive value; LR+ = positive likelihood ratio; LR− = negative likelihood ratio. Proportion confidence intervals are Wilson intervals and likelihood-ratio intervals use the log method; all were calculated using SPSS syntax. Cutoffs and apparent classification estimates are sample-derived. Model 1 = Intermediate + Higher-Cadence Step Ratio; Model 2 = Intermediate + Higher-Cadence Minutes Ratio. Numerators and denominators are shown before percentages for sensitivity, specificity, PPV, NPV, and accuracy. No internal or external validation was performed.

## Data Availability

A de-identified participant-level analytical dataset supporting the main findings has been provided to the editorial office for internal evaluation. Additional participant-level data, a data dictionary, the SPSS syntax used for the MANOVA, Holm adjustment, ROC analyses, and classification calculations, ROC coordinate tables, and 2 × 2 contingency tables may be made available to qualified researchers upon reasonable request, subject to institutional and IRB approval. Raw minute-level StepWatch files and variables not collected or retained are identified in the Section 2 and Section 4.3.
